# Mesh alone versus combined darn and mesh in primary inguinal hernia repair in adults: a randomized control trial

**DOI:** 10.25122/jml-2022-0332

**Published:** 2023-04

**Authors:** Samer Makki Mohamed Al-Hakkak, Ali Abood Alnajim, Alaa Abood Al-Wadees, Mahmood Albo Ahmed

**Affiliations:** 1 Department of Surgery, Faculty of Medicine, Jabir IbnHayyan Medical University, Najaf, Iraq; 2 Department of Surgery, West Suffolk Hospital, Bury St Edmunds, England

**Keywords:** adult hernia, darn repair, primary inguinal hernia, mesh repair, recurrence

## Abstract

Despite the availability of laparoscopy, open surgery remains the most common practice for primary inguinal hernia repair in general surgery. This study aimed to evaluate the combined mesh and darn (CMD) repair compared to mesh alone (MA) repair in treating adult inguinal hernias regarding recurrence and postoperative complications. We conducted a prospective randomized study, including 330 patients with primary inguinal hernias who underwent primary inguinal hernia repair at our facilities between February 2015 and January 2018. Time spent in the hospital, time to resume regular activities, postoperative sequelae, and recurrence rates were assessed. Patients were randomly assigned to 2 groups: CMD repair was performed on 165 patients (Group 1), and MA repair was done on 165 patients (Group 2). Patients were monitored for three years. The average operation time for MA was 62.2 minutes, compared to 72.9 minutes for CMD. The average time to return to normal work was comparable for both groups at around 3 weeks. In Group 2, 12 (7.1%) patients experienced postoperative complications and 3 (1.7%) recurrences. In the CMD repair group, 13 (8.1%) patients had postoperative complications, but no recurrences were observed. Hospitalization duration and postoperative pain were similar between the two groups. At the three-year follow-up, the CMD repair demonstrated a lower recurrence rate than MA, while both groups had similar postoperative complications, hospital stays, and return to normal activities. The operative time was slightly longer for CMD repair compared to MA repair.

## INTRODUCTION

Inguinal hernia repair is the most common surgery in general surgery, with 80% of all hernia repairs in adults being for inguinal hernias [1]. While open inguinal hernia repair is the utmost popular approach, it is unclear which surgical procedure is the most efficient for repairing inguinal hernias in males [1]. Recurrence is a significant challenge after hernia repair, and the use of mesh implantation for reinforcement of the abdominal wall has been explored [1].

The optimal management method for achieving the best results in treating inguinal hernias is still being studied, as inguinal hernias are prevalent in adult populations, with between 3% and 4% of men suffering from this condition [2]. Multiple processing methods have been employed since Bassini came up with a method in 1887, with the primary objective being to achieve lower levels of recurrence and complications, shorter hospital stays, and a quicker return to routine activities [3].

The darn repair was originally described as a tension-free repair, bypassing the shortcomings of conventional herniorrhaphies procedures [4]. Many modifications have been made to the original repair to improve results, and the use of mesh has become more common, particularly in developing or low-income countries where costs are a significant concern [5,6].

In this study, we compared the effectiveness of two repair-free tension mesh repair techniques for primary groin hernias: combined mesh and darn techniques (CMD) and mesh repair alone (MA). We modified the darn repair to create a skeleton-like gridiron to decrease the recurrence rate to almost zero. Our study adheres to the CONSORT criteria [7].

## Material and methods

### Study design and participants

This prospective randomized comparative study was performed at the Department of General Surgery, Al-Sader Medical City, City of Najaf, Iraq, from January 2015 to February 2018. A total of 330 men patients, aged 18-70 years, with primary hernias of the inguinal region classified as Gilbert class three or four were enrolled. Patients were randomly assigned to 2 groups: Group I (n=165) received combined mesh and modified darn repair (CMD), and Group II (n=165) received mesh alone repair (MA). The study was registered at ClinicalTrial.gov (ID: NCT04891601).

### Sample size calculation

**Figure 1:**
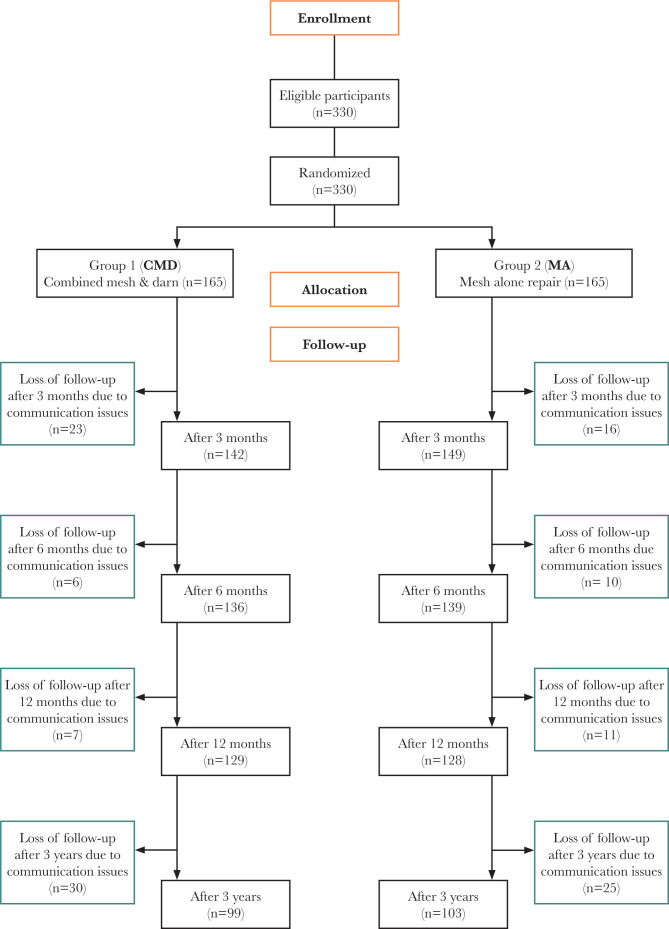
CONSORT 2010 Flow diagram

The sample size calculation for this study was based on an alpha (α) value of 0.05 and power of 0.80, with p-values of 0.2 and 0.05 assumed for p-value one and p-value two, respectively, for chronic postsurgical pain at 6, 24 hours, three, six, and twelve months [8]. The study included two groups, with an initial minimum required group size of 99 individuals and a total of 202 patients enrolled. To account for an anticipated loss of 30% to 40% of patients during the 12-month follow-up period, 330 patients were enrolled at the Department of General Surgery at Al-Sader Medical City, Najaf City, Iraq, between January 2015 and February 2018 (Figure 1). Eligibility criteria for the study required male participants aged 18 to 70 with primary inguinal hernia designated Gilbert class three or four [9]. The study design maintained an alpha (α) value of 0.05 and power of 0.80, with assumed p-values of 0.2 for Group 1 and 0.05 Group 2.

### Preoperative preparation

All patients underwent a comprehensive medical history and clinical assessments, and any predictors, like chronic constipation and cough, were managed prior to the surgery. Ongoing investigations were conducted as necessary, and all patients were required to provide informed consent. Patients with direct or indirect groin hernias were randomized into 2 groups using 330 envelopes, which were fully sealed and mixed. The medical staff performing the surgeries were blinded to group allocation and selected an envelope immediately prior to the operation.

### Surgical procedures

All surgeries were performed by one team of experienced consultant general surgeons specializing in open surgical inguinal hernia procedures. The anesthesia type (general, epidural, or spinal) was determined by anesthetists based on the overall health status of the patient and specific issues or problems. A single ceftriaxone dose (1g IV) was administered preoperatively. In patients with direct inguinal hernias, the fascia transversalis plication was initially performed using an absorbable 2-O polylactic acid suture. In cases of indirect inguinal hernia following sac excision, either the CMD or MA repair was performed.

### Modified darn repair

**Figure 2:**
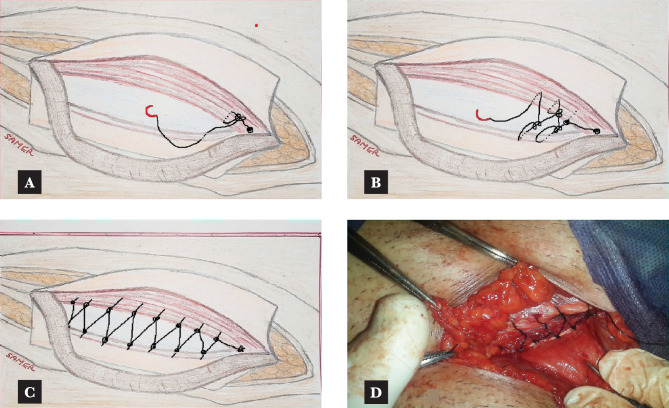
Modified darn procedure.

**Figure 3:**
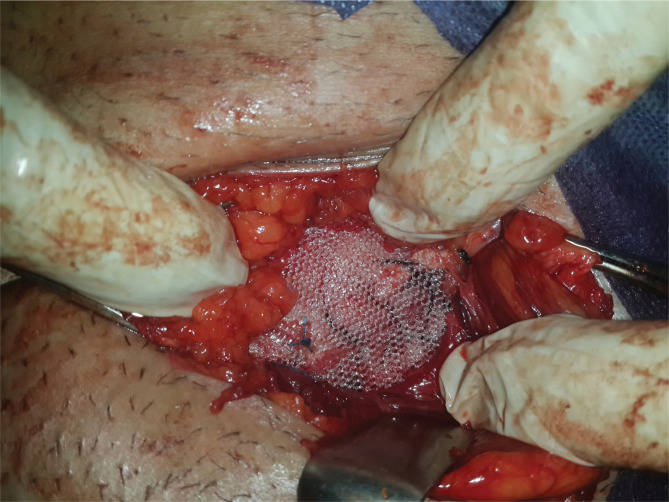
Combined darn and mesh repair.

The modified darn repair procedure began with a suture on the pubic tubercle using nylon O. The suture went through the conjoint tendon from medial to lateral, creating a double loop in a manner that is free of tension. The suture was then passed through the inguinal ligament from its medial side, creating another double loop in a manner that is free of tension. This process continued in a medial to lateral direction, forming a grid-like pattern with two centimeters of space between each stitch (Figures 2 and 3). When passing sutures around the cord, the surgeon exercised high caution and precision to pass the stitches near the cord at the deep ring without applying pressure. The stitches were then buried on the deep ring medial side.

### Mesh repair

The mesh repair procedure followed the technique mentioned by Kingsnorth et al. [10]. An 11× 6 cm prolene mesh was used during the repair.

### Outcome measures

Primary endpoints included

1. Chronic post-surgical pain, assessed by the surgeon during outpatient visits using the modified Visual Analogue Scale (VAS) [11] at 6 and 24 hours and 3, 6, and 12 months postoperatively.
2. Recurrence rates determined at 3, 6, 12 months, and 3 years during follow-up examinations at the outpatient clinic.
3. Duration of operation (in minutes), recorded by the medical staff during the procedure.
4. Early postoperative complications (urinary retention, elevated testis to upper scrotum, seroma, infection) assessed during examinations and outpatient care follow-up.
5. Time to resume household and professional activities (in days), as reported by the patients and evaluated by the surgeons during outpatient follow-up visits on day 14 post-surgery.

### Statistical analysis

Data were reviewed using SPSS 17.0. To compare chronic post-operative pain levels (measured by the modified visual analog scale) between two groups, Student’s t-test was performed at 6 and 24 hours, as well as at 3, 6, and 12 months postoperatively, both at rest and during motion. Initial assessments were based on comparative data at 6 and 24 hours and at 3, 6, and 12 months postoperatively. To ensure that the overall significance level remains at 0.05, the level of significance for the t-test was adjusted to 0.025 after applying the Bonferroni correction.

## Results

Patient demographic and clinical data, including body mass index (BMI), age, smoking status, duration of complaints, affected side, and categorized as having Gilbert, showed no significant differences between the two groups (Table 1). The average operating time for CMD (Group 1) was significantly longer than for MA (Group 2). Despite the expectation of potential variations between the two groups, the study results indicated no significant differences in terms of the intravenous analgesic dose required on the day of surgery or during hospitalization (Table 2). Patients who underwent CMD (Group 1) returned to home activities after 1.20 ± 0.39 days and business activities after 6.79 ± 1.11 days, while patients who underwent MA (Group 2) returned to home activities after 1.20 ± 0.40 days and work activities after 6.70 ± 1 days (Table 2). There were no significant differences observed in these measures between the two groups.

**Table 1:** Patient demographics/clinical data.

	Group 1 (CMD), n=165	Group 2 (MA), n=165
**Age**		
**Mean**	37.47	37.44
**Range**	20–59	20–59
**SD**	11.97	11.93
**BMI**		
**Mean**	26.44	25.69
**Range**	24.8–27.9	23.8–27.9
**SD**	1.10	1.16
**Smoking**		
**Smoker**	43 (40.2%)	38 (31.9%)
**Nonsmoker**	26 (24.3%)	42 (35.3%)
**Ex-smoker**	39 (36.4%)	39 (32.8%)
**Duration of complaints (measured in months)**		
**Mean**	27.12	21.94
**Range**	0.75–192.0	2.0–192.0
**SD**	45.1	42.79
**Affected sides**		
**Right**	81 (81.8%)	79 (76.7%)
**Left**	28 (28.2%)	24 (23.3%)
**Type of hernia**		
**Direct**	109 (66.6%)	112 (67.7)
**Indirect**	56 (33.4)	53 (32.3)

**Table 2:** Post-operative and operative data

	Group 1 (CMD), n=165	Group 2 (MA), n=165	t	P
**Time of operation (min)**				
**Mean**	72.90	62.20	**2.469**	**0.014**
**Range**	45–120	60–90		
**SD**	12.80	19.89		
**Hospital stay (day)**				
**Range**	1–2	1–2	1.557	0.122
**Mean**	1.03	1.08		
**SD**	0.20	0.30		
**Return to regular activity (day)**				
**Range**	6-9	6-9	2.55	0.13
**Mean**	6.84	6.67		
**SD**	0.22	0.27		
**Dose of analgesia (mg)**				
**Mean**	79.91	78.78	0.485	0.628
**Range**	75–150	75–150		
**SD**	18.63	16.48		

Bold values indicate statistical significance (P<0.05).

Initial postoperative complication rates did not show significant differences between the CMD group (Group 1) and the MA group (Group 2). In Group 1, five patients experienced wound infection, compared to seven patients in Group 2. Conservative treatment was administered to all these patients with wound care and intravenous antibiotics. Seroma was observed in 4 patients in Group 1 and seven patients in Group 2, and all cases were successfully managed by aspiration using sterile techniques (Table 3).

**Table 3:** Initial postoperative sequels.

	Group 1 (CMD)		Group 2 (MA)		Fisher exact Two-tailed P-value*
	No.	%	No.	%	
**Initial complications: (within one month postoperative)**					
**Retention of urine**	6	3.6	5	3	0.867
**Seroma**	4	2.4	7	4.2	0.952
**Early infection**	5	3	6	3.6	0.223
**Testicular elevation to the neck of the scrotum**	1	0.9	0	0.0	0.655

No statistically significant differences (P>0.05) between the groups.

All patients were followed up at three-, six-, and twelve months post-surgery, although some patients were lost to follow-up over time. At twelve months, 129 patients in Group 1 and 128 in Group 2 remained in contact. Regarding late complications, only one patient experienced late wound infection six months after MA surgery. This patient, who had uncontrolled diabetes, was managed with pus drainage without mesh removal and was successfully treated with antibiotics. Recurrence occurred in three patients in Group 2, while no recurrences were observed in Group 1, demonstrating a statistically significant difference.

In the study, the Visual Analog Scale (VAS) [11] was used to measure the pain experienced by patients following surgery, both at rest and with movement, during both the immediate and long-term postoperative periods. The results are presented in Table 4. No significant differences in pain levels were observed between CMD and MA during the early and late postoperative periods. Stiffness sensation was also similar between the two groups (P=0.1), with 6% (6 out of 99 patients) of CMD patients and 6.7% (7 out of 103 patients) of MA patients reporting stiffness.

**Table 4:** VSA values during the initial and late postoperative period.

	Group 1 (CMD)	Group 2 (MA)	t	P
**I. Rest**				
**VAS at 6h after operation**				
**Number**	165	165	**3.795**	**0.1**
**SD±Mean**	32.7±11.22	42.5±24.5		
**VAS one day after the operation**				
**Number**	165	165	**5.902**	**0.1**
**SD±Mean**	25.5±9.90	34.3±11.84		
**VAS three months post operation**				
**Number**	165	165	**4.488**	**0.1**
**SD±Mean**	16.57±8.24	22.73±10.5		
**VAS six months post operation**				
**Number**	165	165	1.185	0.240
**SD±Mean**	9.84±8.65	11.64±11.3		
**VAS twelve months post operation**				
**Number**	165	165	**9.183**	**0.1**
**SD±Mean**	1.24±2.34	5.13±3		
**II. With movement**				
**VAS one day after surgery**				
**Number**	165	165	**6.719**	**0.1**
**SD±Mean**	39.8±22.96	58.91±19.8		
**VAS three months post-surgery**				
**Number**	165	165	**7.524**	**0.1**
**SD±Mean**	22.9±11.64	38.1±16.27		
**VAS six months post-surgery**				
**Number**	165	165	**6.710**	**0.1**
**SD±Mean**	18.21±3.64	24.9±8.7		
**VAS twelve months post-surgery**				
**Number**	165	165	**10.195**	**0.1**
**SD±Mean**	3.64±2.90	8.97±3.70		

The bolded values demonstrate statistical significance, with a significance level of P<0.05.

## Discussion

The evolution of surgical techniques for inguinal hernia repair and technological advancements has led to a surge in innovation and progress. Historically, the recurrence rate of conventional herniorrhaphies may have been attributed to tension generated along the sutured line. Consequently, Lichtenstein (1920-2000) introduced non-tension repair, which has gained widespread acceptance as the preferred method for open hernia repair [10]. With the extended use of biomaterials in inguinal hernia treatment, complications have paved the way for new directions in surgical procedures [10]. Furthermore, factors such as recurrence, postoperative chronic pain, time to resume activities at work and home, and infection rates serve as indicators of surgical success.

The primary objective of the present study was to compare the outcomes of combined darn-mesh repair (CMD) with mesh alone (MA). Although the mean operating time for CMD was notably longer than that for MA, this may be attributed to the learning curve associated with the initial CMD surgeries. We observed that the surgical team, despite performing both techniques, was able to complete the surgery faster, around 10-15 minutes, with one of the methods, which we believe is acceptable if it reduces or prevents recurrence. Both CMD and MA patients had similar return-to-work outcomes. However, an additional CMD operation time of 10 to 15 minutes is acceptable if they prevent recurrent procedures.

Recurrence rates are an important factor in hernia assessment. In the past, tissue repair involved approximating the edges of the abdominal fascia and muscles with permanent prolene sutures, which unfortunately placed considerable tension on the wound, potentially leading to complications such as wound dehiscence, ischemic tissue damage, and recurrence [10]. CMD offers a tension-free dual repair that prevents edge ischemia, possibly explaining the positive CMD outcomes observed in this study, which reported no recurrences during the three-year follow-up period. Previous studies have reported variable recurrence rates [12,13]. CMD prevents mesh dislocation by evenly distributing tension force across a larger surface, thereby reducing recurrence rates compared to MA. In the current study, three recurrences were documented in the MA group (Group 2), including two direct and one indirect hernia.

Postoperative pain and discomfort associated with hernia repair have been linked to tissue damage, nerve injuries, and biomaterials [14]. In our study, the MA group reported similar postsurgical VAS scores during periods of rest and physical activity compared to the CMD group. An intense inflammatory response triggered by the prolene thread used in MA, as opposed to CMD, may result in scarring, stiffness, and reduced flexibility of the wall of the abdomen, as well as shrinkage of the biomaterial over time, potentially explaining the discomfort associated with MA [15]. There is a notable similarity in stiffness between MA and CMD.

One patient experienced a late, deep-seated infection following the MA technique, which required drainage and antibiotic coverage, but mesh removal was unnecessary. Inguinal hernia repair with the use of mesh in hernia repair is generally considered a clean surgical procedure, and the occurrence of postoperative infections is estimated between 2.0% and 4.2 [16, 17]. Mesh-related infections are influenced by the foreign body reaction, which depends on the amount of prosthesis (mesh) used [18]. Surgeons should aim to reduce the mesh surface area implanted during hernia surgery to minimize bacterial colonization [18]. The modified CMD technique requires a smaller mesh size for hernia repair. The modified darn creates a tension-free approximation between the conjoint tendon and inguinal ligament, potentially reducing mesh infection and seroma rates [19].

We believe follow-up time plays an important role, and the short follow-up time may have limited our study. However, after a monitoring period of three years, CMD seems to be similar or equivalent to MA, with similar chronic postoperative pain and a lower recurrence rate. To corroborate our findings, more patient data and longer follow-up periods are necessary.

Several previous studies have compared tension-free hernia repair techniques, reporting similar outcomes regarding postoperative pain levels and the resumption of daily activities, and recurrence rates [19]. Unfortunately, in the past, the cost of mesh was very expensive for low-income countries, limiting the use of both techniques. However, the price of mesh has significantly decreased, making it more affordable and now commonly utilized in most inguinal hernia surgeries.

In this study, we adopted a modified mesh with a grid-like structure to provide increased stability and allow more time for fibrosis induction, thereby preventing recurrence due to the delayed fibrous formation that can occur under high pressure in the anterior abdominal wall. This dual-acting, tension-free approach may be particularly beneficial for direct inguinal hernias. Interestingly, our research documented a recurrence rate for indirect inguinal hernias in Group 2 (MA), further supporting the adoption of CMD for all primary inguinal hernias, especially direct ones.

The primary limitation of our study is the monitoring of patient progress over time, as many patients are typically lost to follow-up after three years, making it difficult to assess late recurrence rates in relation to the modified techniques. Another limitation is the relatively small sample size. To validate the effectiveness of the new modified technique in preventing early recurrence rates, larger, multicenter, randomized trials are necessary. The primary findings indicate that the combined darn repair offers additional support in treating primary inguinal hernias, especially direct ones. Further randomized trials and larger multicenter studies are needed to obtain more rigorous and conclusive evidence supporting the superiority of CMD over MA. In this study, 66 patients from Group 1 and 62 patients from Group 2 were lost to follow-up. It is often difficult to maintain contact with patients who have recovered well post-surgery and have not experienced complications or recurrence, resulting in the loss of connection after more than one year.

## Conclusion

The modified combined darn-mesh repair demonstrated superior efficacy in reducing recurrence rates compared to mesh alone in managing primary adult inguinal hernias, particularly in cases of direct inguinal hernias. Nevertheless, both techniques yielded comparable outcomes regarding postoperative pain and the resumption of regular activities.
